# Big Data Approaches in Heart Failure Research

**DOI:** 10.1007/s11897-020-00469-9

**Published:** 2020-08-12

**Authors:** Jan D. Lanzer, Florian Leuschner, Rafael Kramann, Rebecca T. Levinson, Julio Saez-Rodriguez

**Affiliations:** 1 Institute for Computational Biomedicine, Bioquant, Heidelberg University, Faculty of Medicine, and Heidelberg University Hospital, Heidelberg, Germany; 2 Faculty of Biosciences, Heidelberg University, Heidelberg, Germany; 3 Internal Medicine II, Heidelberg University Hospital, Heidelberg, Germany; 4 Department of Cardiology, Medical University Hospital, Heidelberg, Germany; 5 DZHK (German Centre for Cardiovascular Research), Heidelberg, Germany; 6 Department of Nephrology and Clinical Immunology, RWTH Aachen University, Aachen, Germany; 7 Department of Internal Medicine, Nephrology and Transplantation, Erasmus Medical Center, Rotterdam, The Netherlands; 8 Joint Research Centre for Computational Biomedicine (JRC-COMBINE), Faculty of Medicine, RWTH Aachen University, Aachen, Germany

**Keywords:** Heart failure, Big data, Omics, Single cell, Machine learning

## Abstract

**Purpose of Review:**

The goal of this review is to summarize the state of big data analyses in the study of heart failure (HF). We discuss the use of big data in the HF space, focusing on “omics” and clinical data. We address some limitations of this data, as well as their future potential.

**Recent Findings:**

Omics are providing insight into plasmal and myocardial molecular profiles in HF patients. The introduction of single cell and spatial technologies is a major advance that will reshape our understanding of cell heterogeneity and function as well as tissue architecture. Clinical data analysis focuses on HF phenotyping and prognostic modeling.

**Summary:**

Big data approaches are increasingly common in HF research. The use of methods designed for big data, such as machine learning, may help elucidate the biology underlying HF. However, important challenges remain in the translation of this knowledge into improvements in clinical care.

## Introduction

In the past 5–10 years, big data has become an integral part of the study of cardiovascular disease. There are many definitions of big data; however, one definition is data large or complex enough that they cannot be analyzed or interpreted by traditional methods. As a result, computational methods, primarily statistics and machine learning (ML), are used to analyze this data. Several big data technologies are starting to be applied in the clinic: for example, genomics and transcriptomics are used for patient stratification in breast cancer diagnosis and treatment [1, 2] and can be used to determine acute cardiac allograft rejection [3, 4]. However, due to challenges in clinical implementation and questions about the benefits of these methods [5], most big data approaches are implemented in preclinical research.

**Fig. 1:**
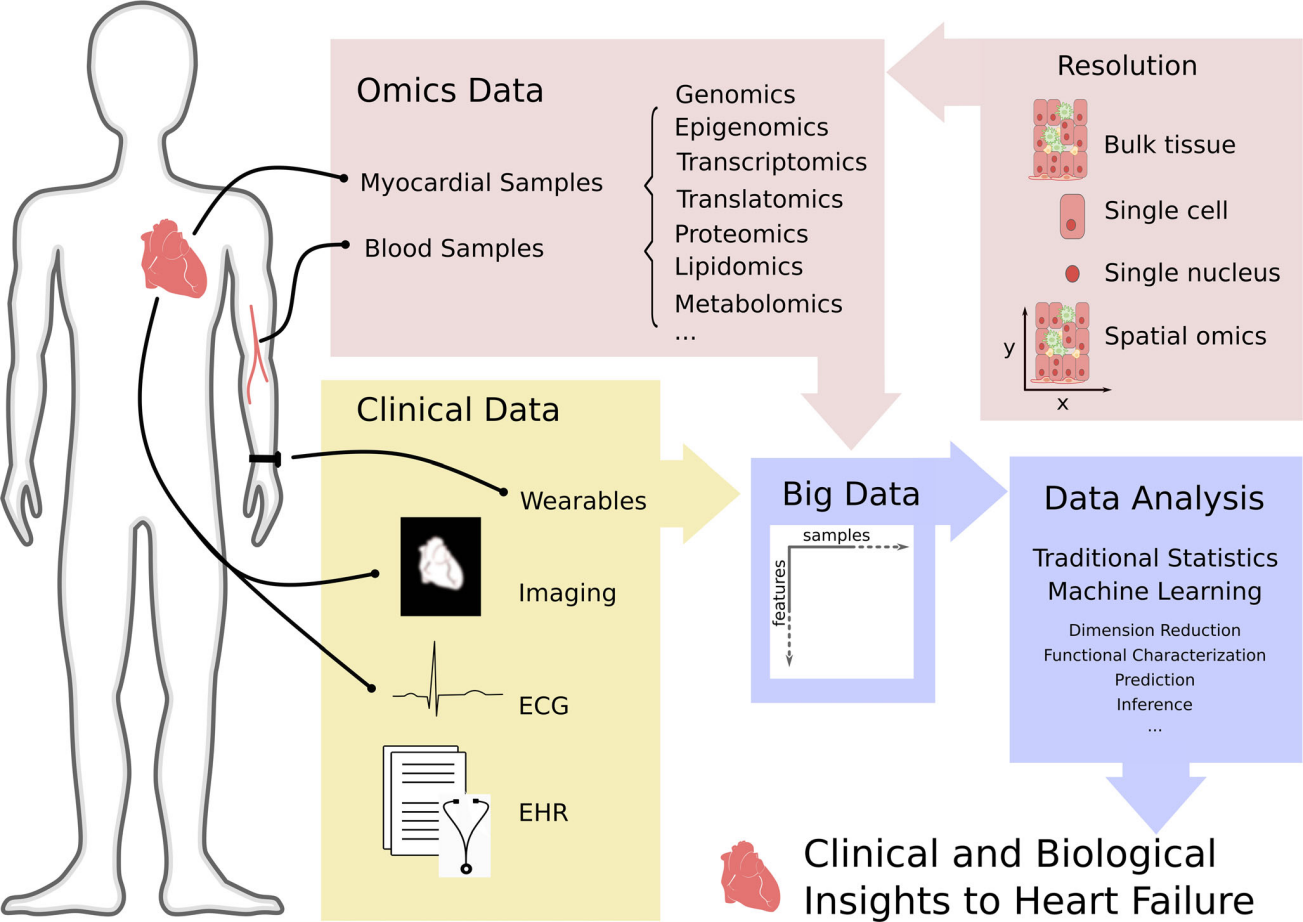
Types of big data in heart failure and the body location from which samples are taken for that data type. Omics and clinical data are the two common big data types to study HF. Clinical data can be gathered via wearables, imaging techniques, echocardiography (ECG), and electronic health records (EHRs). Different omics technologies primarily analyze cardiac tissue or blood and include genomics, transcriptomics, translatomics, proteomics, metabolomics, and lipidomics. Specimen can be studied at different resolutions, including bulk, single cell, single nucleus, and spatial level. To date the different tissue resolutions are not yet available for every omic. Data analysis is challenged by accuracy, structure, and volume of omics and clinical data. Traditional statistical as well as machine learning methods are employed to extract essential information to improve biological understanding and clinical care in HF.

Chronic heart failure (HF) is a prime target for big data research due to the complex etiology of the syndrome, the large number of risk factors, the high degree of comorbidity in patients, and the prolonged and progressive course of disease. Big data used for the study of HF are derived from a variety of sources (Fig. 1). Some of these sources are dependent on tissue such as blood or myocardial samples, while others are ascertained through clinical care or wearable devices.

In this review, we discuss the biological and clinical impact of the application of common big data types and computational approaches across the spectrum of human HF etiologies and subtypes, including inherited and acquired HF as well as HF with preserved and reduced ejection fraction (HFpEF and HFrEF, respectively). While many types of big data are used for the study of HF (Table 1), this review will focus on several areas of omics, including genomics, epigenomics, transcriptomics, and proteomics, as well as big clinical data. We also address several current issues with big data collection and analysis, and reflect on the future of these methods in HF.

**Table 1:** Big data of types in heart failure. Many types of big data used in the study of HF are listed below along with a brief description. Data types specifically addressed in this review are in italics

Types of big data	Description	Examples in HF
*Genomics*		
Genome-wide association study (GWAS)	Observational study testing the association of genome-wide common genetic variation with a trait in a population of individuals.	Reviewed in [6, 7]
Whole-genome sequencing (WGS)	Sequencing of the whole genome. Usually applied in the study of inherited disorders resulting in HF.	[8, 9]
Whole-exome sequencing (WES)	Sequencing of the exome (protein-coding portion) of the genome. Usually used to study forms of HF with known genetic etiologies.	Reviewed in [6]
*Transcriptomics*		
Microarray	Quantification of RNA by fluorescence measurement of cDNA using chips. Limited to genes targeted by array chip.	[10, 11]
Bulk RNAseq	Quantification of RNA though sequencing of cDNA, alignment to reference genome, and counting.	[12, 13]
Single-cell RNAseq	Single cell or nucleus isolation prior to RNAseq	[14••]
Spatial transcriptomics	RNAseq performed on patches of tissue on slides	[15]
*Proteomics*	The study of proteins or peptides in a targeted or agnostic manner.	Reviewed in [16–18]
Metabolomics	The agnostic or targeted study of metabolites.	Reviewed in [19]
Lipidomics	The study of the complete or targeted lipid profile in an individual or population	[20, 21]
Wearables	An item worn externally that provides continuous data on parameters like heart rate, blood pressure, or fitness activity.	Reviewed in [22]
*Clinical data*		
Electronic health records	Electronic data representing patients or patient groups produced for the purpose of managing clinical care	[23]
Imaging data	The process of creating visual representation of physiology. Examples include CT, MRI, echocardiography, EKG, X-ray.	Reviewed in [24]

## Big Data Computational Methods

In order to analyze big data, methods that account for both the size and the complexity of the dataset are required. Data noise, spurious correlations, and limitations in computational power are a few of the challenges that these types of analysis methods are designed to overcome [25]. Big data analysis protocols vary based on the nature of the data collected as well as the specific research question. Nonetheless, there are shared concepts that are common in these analyses that we will briefly discuss: dimension reduction, ML, and a popular branch of ML called deep learning (DL). For more detailed methodological overview, see [26–28].

When a large number of features are measured, data visualization and interpretation is difficult. Thus, feature or dimension reduction is a common aim of computational methods [29]. Data-driven approaches like principal component analysis transform features to a lower dimensional space by finding combinations of features that capture most of the variability in the data. These combined features, however, are often hard to interpret as they are a mathematical combination of arbitrary molecules. Thus, an alternative is to perform dimension reduction based on interpretable features. For this purpose, molecules are grouped into processes such as cellular pathways, based on prior knowledge that is available in databases. There are many methodological approaches that then try to estimate which processes are more or less active, based on the levels of the molecules that belong to them (e.g., gene set enrichment analysis [30]).

After preprocessing and potentially dimension reduction, statistical learning and modeling is often applied to make estimations about populations (inference) or predictions of new experiments. These analyses often rely on ML, a variety of algorithms to carry out computational tasks without detailed instructions provided by the user. ML algorithms can be classified as unsupervised or supervised. Unsupervised algorithms learn underlying patterns from data while supervised algorithms learn from labeled data to perform tasks like classification or prediction. For this, a portion of available data, referred to as the training data, is used to fit a model. The model performance is then assessed on test data, data that has not been used for model training.

A common supervised ML algorithm is a neural network. A neural network is characterized by nodes that are organized in layers, inspired by biological neuronal circuits. Each node can be regarded as a function that processes inputs to generate outputs that become the inputs of the next layer of nodes. The information processing of each node is learned from data by an optimization method that adjusts the node’s function parameters to minimize the error in performing a task, learning from each sample in a data set. Deep learning (DL) is a specific type of machine learning that uses neural networks with many layers. Deep learning is difficult to interpret since information processing via different nodes and layers becomes incomprehensible. However, it is very powerful in performing without human input highly difficult tasks such as interpretation of biomedical imaging or clinical health records that otherwise would require high domain knowledge.

## Omics in Heart Failure

High-throughput methods enable researchers to study molecular profiles of tissues at high resolutions (Table 1). This field is generally referred to with the suffix *-omics*. Omics technologies can be described as non-targeted—those that aim to measure complete molecular profiles in an unbiased manner—and targeted—those that have predefined molecules of interest. While non-targeted omics are often treated as a complete representation of a molecular profile, the biases that underlie these representations need to be considered as possible sources of error. Technical bias often results from favoring abundant or easy to read molecules and can be only partially corrected during normalization procedures before data analysis. As technologies develop, technical biases are addressed (e.g., long-read RNAseq can overcome negative bias towards long transcripts and improves isoform detection [31]). Further biases are introduced at the analysis level, when prior biological knowledge is used to reduce the dimensions of omics data (see “Big Data Computational Methods”). The consultation of prior knowledge can forestall new discoveries by disregarding valuable information. Furthermore, bioinformatic databases are a main source of biological knowledge and their inherent biases and inaccuracies are integrated into analyses that use them.

In HF, the specimen for omics analysis usually is cardiac tissue or blood (e.g., peripheral blood mononuclear cells (PBMCs)). While myocardial omic analyses can help elucidate disease mechanisms and identify biomarkers and therapeutic targets, the tissue availability for human samples is limited. Blood samples are easier to access and can help survey HF patients at a higher temporal resolution. They are used for biomarker detection and to study genomics as well as the role of circulating cells while the origin and pathophysiology of circulating molecules can be difficult to define.

Different omics technologies pose similar challenges on data analysis and evaluation, including problems concerning accuracy, imputation, integration, replication, and interpretation. We will discuss recent advances and challenges in important omics, in particular genomics, epigenomics, transcriptomics, and proteomics.

### Genomics

Genomics is one of the classic areas of big data, studying the role of the genome in disease. Within genomics, analyses are split by technique: genome-wide association studies (GWAS) investigate specific predetermined single nucleotide polymorphisms (SNPs), while whole-exome sequencing (WES) and whole-genome sequencing (WGS) use next generation sequencing to identify all variations in the coding regions or the complete genome, respectively. GWAS is typically used to study common HF, while WES and WGS are more frequently used to study subtypes of HF with known genetic etiologies such as familial dilated cardiomyopathy (DCM) [32]. In both GWAS and whole-genome association studies (WGAS) difficulties include limited information at each single nucleotide polymorphism (SNP), difficulty understanding the biological mechanism that may drive the association for SNPs not in genes, and the need for large populations to gain sufficient statistical power [33].

As a result of years of HF GWAS with limited associations that provided little insight into the biology of the disease [34], recent GWAS have focused on gathering sufficiently large samples, looking at HF subgroups [35], and investigating biomarker and intermediate quantitative traits relevant to HF [7, 36, 37]. While GWAS of HF have been reviewed elsewhere [6] [7], we want to highlight the recent publication of the largest GWAS of HF to date: Shah et al. found 12 independent signals at 11 loci associated with HF risk factors or structural parameters of the left ventricle (LV) [38••]. After statistical analysis to determine causality, several loci that remained as risk factors mapped to genes involved in cellular senescence, cardiac development, and protein homeostasis. These results indicate that while much of the genetics of all cause HF is due to risk factors, other innate biological pathways relevant to cardiac function also play a role in genetic predisposition to disease. However, like much of the GWAS literature, these results are also limited in scope as the population studied only included European ancestry individuals.

### Epigenomics

Epigenomics is the study of alterations across the genome that regulate genome expression and function without altering the DNA sequence. Some of the epigenomic alterations that are known to be relevant in HF include DNA methylation [39], chromatin conformation mapping [40], and histone modifications [41, 42]. Many studies that apply epigenomics also use data from other omics techniques, often either genomics or transcriptomics into a multi-omics approach [39, 43, 44].

Despite these examples, there are many challenges in both the analysis and interpretation of epigenomic data. Different groups use different analysis workflows and there is limited consensus on the best way to analyze data. The epigenome is highly dynamic, meaning that with only a single sample at a single time-point, it can be difficult to determine which changes are causes and which are consequences of the cell state [45]. In data interpretation, the cell and tissue specificity of the epigenomic landscape means that it can be difficult to be certain which changes are relevant to disease state [45, 46]. Despite these challenges, epigenomics provide a natural bridge between knowledge of the genetic state in HF and potential biological consequences.

### Transcriptomics

The transfer from genetic code to cellular function is mediated by the transcription of ribonucleic acid (RNA). RNA can be translated to proteins (coding RNA or messenger RNA), or execute structural (e.g., ribosomal RNA) or gene regulatory functions (e.g., micro RNA, long non-coding RNA). The quantification of the set of RNA molecules (transcripts) produced by the genome is generally referred to as transcriptomics and provides important understanding of disease mechanisms [47]. As transcriptome profiles can cover up to ~ 20.000 coding and ~ 15.000 non-coding genes [48], analysis and functional interpretation is challenging. To extract relevant information from transcriptome data, dimension reduction methods, enrichment based analysis, linear modeling, clustering algorithms and other ML techniques are routinely applied. The combination of these methods with prior biological knowledge constitutes a key concept of functional interpretation of large-scale gene expression data [49]. Among the disadvantages of using bulk transcriptomics is their susceptibility to fluctuations in cellular composition, which can lower sample comparability. One solution to this is computational cell deconvolution methods [50], which calculate cell fractions from bulk measurements and can estimate cell-specific expression profiles [51]. Cell deconvolution of human heart tissue has been performed [52] and might serve as a first example to enhance future HF transcriptome analyses.

The first high-throughput transcriptomic study on myocardial human HF was published in 2000 [53]. In the subsequent decades, technological and bioinformatic advances in transcriptomics have improved our comprehension of cardiac hypertrophy [54], reverse remodeling [55], cardiac metabolism [10, 56], cardiac fibrosis [57], and immune dysregulation [58] in HF. Several studies made their data sets and protocols publicly available on platforms like NCBI’s gene expression omnibus. However, few attempts have been made to compare transcriptomic HF studies [59–61]. The continuing development of sophisticated data analysis methods invites the retrospective re-analysis and integration of published HF studies, although data integration from different platforms, centers, and technologies presents many challenges [62].

Transcriptome study of myocardial remodeling in HF is complicated by tissue accessibility. Thus, for patient safety, most studies analyzed tissue from HF patients undergoing heart transplantation or LVAD treatment, leaving a knowledge gap of gene expression profiling in HFpEF patients. A recent study compared myocardial transcriptomes in patients with clinical profiles suggesting HFpEF with those not displaying signs of HF [63•] gene dysregulations similar to those observed in HFrEF (TNNT2, LUM and p53). Future research is required to specify differences between cardiac remodeling in HFpEF and HFrEF patients to enable development of disease specific therapy, which is currently lacking.

The profiling of non-coding RNA has provided targets for diagnostic and therapeutic purposes in HF [64, 65]. As HF specific non-coding RNAs can be detected in bloodstream, developing miRNA panels to stratify HF patients by prognostic or diagnostic aspects has been a major focus. However, the clinical utility still has to be demonstrated and the (patho-)physiologic role of circulating RNAs remains unclear. Here, a functional microRNA screening approach could help to prioritize candidates [66].

Translatomics can be described as the quantification of translating mRNAs and ribosomes providing important information of subsequent RNA regulation [67]. To address how these layers of gene regulation connect in the failing heart, a study by van Heesch et al. combined genotypes, transcriptomes, and translatomes in 80 hearts (control vs. dilated cardiomyopathy) [13••]. Deciphering regulation in protein biosynthesis, the balance between transcriptional and translational gene regulation was elucidated, for example mitochondrial processes are initiated during transcription and significantly enhanced on the translational level. Furthermore, protein-truncating variants of DCM causing genes were reported to inefficiently terminate translation, providing insights in the pathogenicity of genetic variants. Excitingly, the authors report that circular RNAs (circRNAs) and long non-coding RNAs (lncRNAs) were found to be also translated to novel microproteins.

### Single-Cell RNAseq

With single-cell RNAseq, the transcriptome of individual cells can be measured, providing tissue profiling at unprecedented granularity. Bulk RNAseq fails to account for a functional diversity of cell types that might be crucial in understanding the orchestration of myocardial syncytium in health and disease. Single-cell expression profiles can inform about cell lineage heterogeneity [68], inter cell communications [69], individual transcription factor and pathway activity levels [70], or can be integrated within multi-omic approaches [71].

The main challenges in the application of this technology included separation of single, viable cells and subsequent amplification of a minute amount of RNA. Different approaches to overcome these hurdles vary in gene coverage and multiplexing ability (i.e., the capacity to process in parallel) [72, 73]. The cardiac tissue poses additional challenges. Since cardiomyocytes (CM) are too large for many cell sorting approaches, single nucleus RNAseq can be applied, which involves isolating the nucleus rather than the whole cell prior to sequencing. The transcriptional profile of single cell and single nucleus RNAseq has been reported to be comparable during CM differentiation [74]. As the transcriptional profiles of mono- and polynucleated CM were reported to be similar [75], application of single nucleus RNAseq on cardiac tissue is encouraged. The plethora of information gathered by single cell RNAseq poses new challenges to big data analysis that have only partially been met. These include the need to quantify uncertainty in measurements and efficiently handle gene dropout rates; the limited benchmarking possibilities; the need to scale to higher dimensional data, as more cells and more genes can be measured; and the integration of multiple levels of single-cell omics [76].

Single-cell RNAseq has already been applied to study the cardiovascular system (reviewed in [77–80]). To date, studies have focused on the description of cardiac cell lineage heterogeneity and trajectory in mice [79, 81–83], as well as on human cardiogenesis [84–88].

HF was studied in murine models, investigating cell cycle arrest [89] and adaptive remodeling of CM [90]. Human HF description on single cell level was recently reported [14••]. A total of 21,422 single cells from 14 control and 8 failing hearts were compared. The reported cellular heterogeneity was suggested to reflect functional specializations. Macrophages fulfill very heterogeneous tasks in the human heart [91], which was mirrored by different macrophage clusters, some of which presumably specialize in regulating the conduction system, whereas other clusters displayed traits that suggested involvement in immune response regulation. A cluster of endothelial cells (EC) was characterized to highly express ACKR1. These ECs decreased in HF and might exert cardioprotective functions, as injection of ACKR1^+^ ECs into a murine HF model improved cardiac function compared to control. This study highlighted that cell heterogeneity within lineages relates to functional specialization and pathological condition, aspects that can only be studied with single-cell resolution. The knowledge of such functional cellular subgroups could enable the targeting of such clusters to boost their cardioprotective ability.

### Spatially Resolved Transcriptomics

As single-cell analysis is exhibiting a soaring development, spatially resolved transcriptomics add yet another layer of information and complexity. Study of the single cell or bulk transcriptome does not regard the complex tissue architecture of the heart. Further, many pathological processes like fibrotic scarring or ischemia can be local aberrations that require to be studied within tissue context. Spatially resolved transcriptomics could overcome these hurdles by gathering gene expression data while retaining respective spatial information. Many approaches are limited in gene coverage and/or in spatial resolution [92]. Stahl et al. developed spatial transcriptomics, a technique that compartmentalizes tissue into patches which are subsequently profiled via RNAseq [93] and set the path for further development [94]. Other technologies are under active development to improve the spatial resolution and coverage of genes of these methods [95, 96]. The data generated by these technologies still require a sound computational framework to integrate both layers of information.

In a pilot study, Asp et al. analyzed cardiac fibrotic samples from three HFpEF patients and demonstrated the feasibility of performing spatial transcriptomics on adult human heart tissue [15]. A hallmark study combined spatial transcriptomics, single cell technology and in situ sequencing to create a spatiotemporal map of gene expression patterns during embryonic heart development at an hitherto unseen resolution [85•]. This combinatorial approach is a highly promising field for HF research and is likely to fundamentally improve our understanding of cardiac remodeling in the near future.

### Proteomics

Proteomics is the quantification of proteins, as transcriptomics do not consider the subsequent and frequent regulations of RNA translation or protein modification. Targeted and non targeted proteomic technologies can be applied, differing in protein coverage, protocol complexity and analytical throughput [97]. Data generated by proteomics provides similar challenges to the other omics analysis as discussed above. In HF, proteomics have been applied to unravel protein posttranslational regulation and temporal dynamics [16]. Due to tissue availability, animal models of HF have been more frequently analyzed [18]. In 2017, the first human proteomic heart atlas described 10,700 proteins in 16 anatomical regions, providing a rich resource of known and unknown protein distributions [98].

The plasma proteome of HF patients has been analyzed extensively to identify biomarkers for HF and their genetic risk-association as well as to understand organ crosstalk via blood stream [99–101]. Egerstedt et al. studied the plasma proteome of HF patients in different clinical stages (early HF development, manifest advanced HF, and reversal of HF after heart transplantation) [102••]. They identified 33 candidate proteins that were associated with HF development. The origin of those circulating proteins was investigated by querying public transcriptome and proteome datasets as well as applying spatial transcriptomics on two failing hearts. GWAS was then used to determine genetic loci that were associated with protein candidates. This study demonstrates that the arsenal of omics technologies can be successfully applied to complete biological characterization of candidate biomarkers.

## Clinical Data in Heart Failure

While omics data provides information about the cellular state during disease, how the disease state is viewed and treated in real patients provides additional insight. Clinical data can be described as information about a patient’s health status that is gathered mainly for the purpose of clinical care. These include imaging data, electronic health records (EHRs), and data captured by wearables (Fig. 1). Clinical and omic data types can be analyzed with similar methods; however, they differ regarding their data structure. While omic data are structured measurements, clinical data is often a combination of unstructured, semi-structured, and structured data with the added complication that free text can be subjective or spurious. Thus, clinical data often requires significant pre-processing prior to analysis, a major hurdle for clinical data analysis on a large scale. Highly promising approaches to this challenge of extracting relevant information from unstructured clinical data include natural language processing [103–105], but even structured clinical data is subject to noise resulting from entry errors. Clinical data is frequently sparse, subject to care utilization and documentation habits, and biased, in that health states outside of clinical encounters are rarely reported. Once preprocessing challenges are overcome, clinical data analysis is often subjected to similar statistical and mathematical modeling as omics data for predictive or inference purposes. In HF, patient outcomes have been associated with the presence of a wide variety of comorbid conditions and ejection fraction sub-group. Despite this, mortality and risk of rehospitalization in HF patients remains high. As a result, three major trends have emerged in the use of clinical data for the study of HF: sub-phenotyping, deep phenotyping, and imaging data.

The emergence of sub-phenotyping has caused a shift from the tendency to view HF patients as a single population (or as two clearly defined populations) towards the tendency to view them as a large heterogeneous supergroup composed of many smaller and potentially unknown subgroups [106, 107]. Predicting the outcomes of HF patients, especially within subgroups, is a major area within big data studies using EHR data or other data relevant to clinical care [108]. Adler et al. were able to divide HF patients into those at high and low risk of death based on clinical variables, and their classifier had a better predictive power than any of the individual classifier components, and better than other comparison markers including NT-proBNP [109]. Ahmad et al. divided a group of HF patients into four clusters which differed in age, sex, clinical measures, and comorbid conditions, before building a classifier to predict survival. They found that cluster membership had a modest predictive ability, but performed better than left ventricular ejection fraction alone as the gold standard measure of cardiac function [110]. Other studies have tested multiple types of algorithms for predicting outcomes including HF hospitalization and mortality among HFpEF patients [111], and phenogrouped HF patients who had been randomized to cardiac resynchronization therapy with a regular or implantable cardiac defibrillator prior to evaluation of the effect on HF events and death [112].

Deep phenotyping—the characterization of a phenotype through the comprehensive evaluation of components and intermediate manifestations—has resulted in the use of many diverse types of data. Data including echocardiography [113], electrocardiography (ECG) [114], cardiac magnetic resonance imaging (MRI) [115], tissue imaging [116], implantable monitors [112], and other wearable and non-invasive cardiac monitors [117, 118] are used in combination with ML and DL methods for the prediction and monitoring of HF patients. Laboratory values and intermediate phenotypes are also widely analyzed. The diversity of data types used for the study of HF is rapidly expanding. Analyzing populations that have multiple data in the same individuals can provide detailed information about the progression of disease as well as insights into clinical characteristics that may indicate negative outcomes.

Imaging data constitutes a major branch of big data analysis, facilitating automated assessment of echocardiography, computed tomography, magnetic resonance imaging, and nuclear imaging results. The rise of imaging data from clinical care has happened partially due to improvements in the ability of ML analysis methods for this data. As DL approaches are especially useful to consider the vast amount of features in raw images and integrate those with other clinical variables. For a detailed discussion of these topics, we point the reader to dedicated reviews [24, 119]). However, as with other big data types, important limitations remain. Most importantly, the lack of interpretability of DL models based on image data is a major obstacle for relevance to clinical care.

As a whole, big data from clinical populations has provided great insight into the true phenotypic diversity of HF and has begun to provide links between that diversity and patient outcomes. However, despite the increased understanding of phenotypic heterogeneity, there is still a significant amount to be learned about the relationship between sub-phenotypes and outcomes. While this is a rapidly expanding field, questions about the necessary manual curation of certain data types, inconsistencies in imaging between clinical sites, and privacy concerns remain. The promise of the interface between large scale clinical imaging and electronic health records holds great promise.

## Conclusions

“Since we can never know all the factors that a problem entails, we can never solve it. [..] To arrive at the truth we would need more data along with the intellectual resources for exhaustively interpreting the data.” - Fernando Pessoa, from *The Book of Disquiet* (translated by Richard Zenith)

Despite advances in the use of big data in HF, we are still learning how to use this information to understand the complexities of HF. To date, many challenges remain, as reflected by high mortality and morbidity rates and limited treatment options. However, the direction that HF research has taken towards big data science promises to advance our knowledge substantially. Relevant data is being collected and analyzed in larger numbers with emerging data types forthcoming. Those include image data, wearable data, environmental data and data generated by cardiac monitors such as CardioMEMs [120]. And, the combination of multiple data types, either clinical and molecular data or multi-omic integration is becoming more common. However, despite the innovation in big data and HF, unresolved questions remain.

Data storage and sharing are key aspects of big data research and security breaches on patient data can lead to serious person rights infringements. While research data is often anonymized to protect participants, data re-identification is a serious threat. To minimize the risks of infringing on participant privacy, different data sharing strategies including open consent, controlled access and registered access are used [121].

Among omics data, genomic information has highest re-identification risk and thus elaborate sharing regulations are needed [122]. Approaches include sharing only the subset of data that is not sensitive, sharing only more common genetic variants that will be less specific to a single individual, and requiring strict protocols for data access. Another approach are search engines for genomic mutations where allelic information can be queried with no reference to a patient [123]. Other omic data in general are less specific to an individual, and data is made public on servers like the gene expression omnibus. However, even transcriptome data can be used to infer genetic structural variants and thus facilitate re-identification [124].

Clinical data is highly sensitive and its use in big data research is subjected to strict regulations concerning data privacy and security. Databases like UK Biobank or dbGAP provide clinical and molecular profiles of participants at great depth. Here, controlled access has to be requested by scientists with a research proposal and an agreement to a data handling framework. Most databases store data and regulate access in a centralized fashion, which constitutes a weak point for security breaches as frequently reported in the US [125]. Methods that rely on decentralized networks, such as blockchain, have been suggested to provide additional security and data ownership for individuals [126–128]. However, as with all data security measures, there remains a trade-off between affordable protection and making data sufficiently accessible to researchers. Beyond critical aspects of data safety, costs of storage, energy consumption, and environmental consequences need to be considered.

One of the major issues across big data domains are biases in the collection and analysis of that data. As we rush to collect ever larger sample sizes, we should pause to carefully consider whether we are merely enthralled by ever increasing data samples (so-called data chauvinism [129]) or whether the biological question is best answered by data of the type and quality available. For many omics technologies, the number of features considered requires large samples, or the noise introduced will result in inferior model fitting. In other cases, a large sample size can be less informative if the sampling is of lower quality, for instance if non-probabilistic sampling was applied [130]. Thus, many omic studies, especially those analyzing sparse myocardial tissue, suffer from small patient cohorts that cannot compensate for the biological and clinical variability. A large-scale effort to acquire and comprehensively characterize relevant tissue samples with a variety of omics techniques would ameliorate this issue and potentially provide great insight into the biology of HF. Such efforts have proven valuable in other areas, most notably in oncology. In clinical data analysis we must balance the desire to find subsets of patients that share characteristics, with the goal of making sure that all patients benefit from the potential of precision medicine. Concerns about sampling bias, data missingness, and measurement error in big data, and especially big clinical data, are all relevant to research in HF [131–133]. These data quality concerns are also important because they will directly affect the output of machine learning analyses [134].

Lastly, despite the excitement about big data, the ultimate goal in medicine must always be to improve human health. Physicians should receive additional training allowing them to appropriately evaluate the potential of big data in clinical care [135]. To successfully implement precision medicine approaches based on big data technologies, clinicians will need to understand the strengths and weaknesses of methodologies and have confidence in their relevance to disease. The role of big data in HF prevention and treatment necessitates a multi-disciplinary discussion where physicians are needed to take a leading role.

A significant amount of big data has been generated and analyzed for the study of HF to promote a digitalization of medicine [136] and are adapted to deal with the particular problems posed by HF biology on the various levels that have been discussed. However, challenges still lie ahead. Some are data governance issues, such as patient privacy as well as data access and sharing, while others are more biological or technical, such as integration of multiple data types to describe HF from different perspectives. As the amount of big data generated by different methods continues to accrue, we must piece the biology together, and harness that knowledge to benefit patients. While a unified theory explaining complete clinic and biology of HF might still be unattainable, the era of big data analysis helps us to consider more and more factors and thus brings us much closer to the goal of treating the right patient with the right treatment at the right time.
